# My patient has received fluid. How to assess its efficacy and side effects?

**DOI:** 10.1186/s13613-018-0400-z

**Published:** 2018-04-24

**Authors:** Xavier Monnet, Jean-Louis Teboul

**Affiliations:** 1 Hôpital de Bicêtre, Service de Réanimation Médicale, Hôpitaux Universitaires Paris-Sud, 78, rue du Général Leclerc, 94270 Le Kremlin-Bicêtre, France; 2 Université Paris-Sud, Inserm UMR S_999, 94270 Le Kremlin-Bicêtre, France

**Keywords:** Fluid responsiveness, Pulse pressure variation, Passive leg raising, Fluid challenge, Cardiac output, Extravascular lung water

## Abstract

Many efforts have been made to predict, before giving fluid, whether it will increase cardiac output. Nevertheless, after fluid administration, it is also essential to assess the therapeutic efficacy and to look for possible adverse effects. Like for any drug, this step should not be missed. Basically, volume expansion is aimed at improving tissue oxygenation and organ function. To assess this final result, clinical signs are often unhelpful. The increase in urine output in case of acute kidney injury is a poor marker of the kidney perfusion improvement. Even if oxygen delivery has increased with fluid, the increase in oxygen consumption is not constant. Assessing this response needs to measure markers such as lactate, central/mixed venous oxygen saturation, or carbon dioxide-derived indices. If tissue oxygenation did not improve, one should check that cardiac output has actually increased with fluid administration. To assess this response, changes in arterial pressure are not reliable enough, and direct measurements of cardiac output are required. In cases where cardiac output did not increase with fluid, one should check that it was not due to an insufficient volume of fluid administered. For this purpose, volume markers of cardiac preload sometimes lack precision. The central venous pressure, in theory at least, should not augment to a large extent in fluid responders. The worst adverse effect of fluids is the increase in the cumulative fluid balance. In patients with acute respiratory distress syndrome (ARDS), the risk of aggravating pulmonary oedema should be systematically assessed by looking for increases in extravascular lung water, or, more indirectly, increases in central venous or pulmonary artery occlusion pressure. In ARDS patients receiving fluid, one should always keep in mind the risk of inducing/aggravating right ventricular dilation, which should be confirmed through echocardiography. The risk of increasing the intra-abdominal pressure should be carefully sought in patients at risk. Finally, fluid-induced haemodilution should not be neglected. Like for any drug which has inconsistent effectiveness and may exert significant harm, the correct fluid management should include a cautious and comprehensive assessment of fluid-induced benefits and side effects.

## Introduction

Despite its trivial aspect, administration of fluid in critically ill patients poses several complex problems. Many efforts have been made to determine, before giving fluid, whether it will increase cardiac output. Nevertheless, after fluid administration, like for any treatment which has inconsistent effectiveness and which might induce significant side effects, it is essential to ask: was volume expansion effective? Was it harmful? Fluids are drugs. Assessing their efficiency and their adverse side effects should be part of good clinical practice. It seems to us that this step is often missed. In this review, based on the published data, we will attempt to show how essential it is to carefully evaluate the positive and negative effects of volume expansion once it has been administered and which evaluation criteria might be useful for this purpose.

## Was volume expansion efficient?

### What is fluid efficiency?

**Fig. 1:**
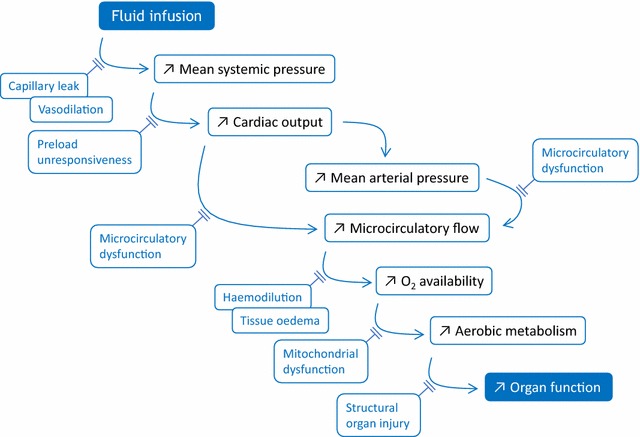
Schematic pathway through which fluid administration leads to organ function improvement, and the issues that may interrupt it

When fluid is administered in case of acute circulatory failure, it is with the final goal of increasing tissue oxygenation and, if it was previously impaired, of improving organ function. Nevertheless, between volume expansion and the resolution of organ failure, multiple steps are to be crossed (Fig. 1). At each one, issues might explain why fluid administration is eventually not effective.

Basically, fluid is administered in order to increase the mean systemic pressure, which is the forward pressure of venous return. Nevertheless, capillary leak or vasodilation may impede that fluid actually increases the stressed blood volume [1]. If it occurs, the raise in mean systemic filling pressure leads to an increase in cardiac output if both ventricles are preload dependent. In such a case, the right atrial pressure does not change or increases to a lesser extent than the mean systemic filling pressure, so that the pressure gradient of venous return increases [2]. If it happens, the increase in cardiac output leads to the increase in the oxygen delivery, i.e. the flow of oxygen that is sent towards the tissues, though the fluid-induced haemodilution tends to smoothen this effect [3]. The increase in cardiac output might increase the mean arterial pressure (Fig. 1). Nevertheless, this is not constant, since the sympathetic system physiologically tends to maintain the mean arterial pressure constant when cardiac output varies [4].

The increase in arterial flow and/or pressure increases the blood flow through the microcirculation and, sometimes, this is accompanied by a significant increase in the proportion of perfused vessels [5]. This step might be impeded by microvascular failure, especially in case of sepsis [5]. Provided that microcirculation is intact, the increase in microcirculatory flow leads to an increased oxygen availability for the tissues, which might be attenuated by tissue oedema. The aerobic metabolism increases in response. Nevertheless, mitochondrial dysfunction, as it may occur during sepsis, might explain why tissue hypoxia persists in spite of increased oxygen availability [6] (Fig. 1).

Finally, the reduction in tissue hypoxia should improve organ function. Nevertheless, this becomes impossible if organ function has been structurally injured by prolonged hypoperfusion. For instance, tubular necrosis explains that acute kidney injury persists in spite of resolution of the acute phase of shock (Fig. 1).

### Did tissue oxygenation improve?

Basically, the decision to infuse fluids should be triggered by obvious signs of tissue hypoperfusion. In particular, it should never be taken only for increasing cardiac output, for instance, on the basis of positive tests of fluid responsiveness. The harmful effects of fluid infusion are today clearly demonstrated, and strategies aiming at systematically maximising oxygen delivery and cardiac output have been shown to be useless or even deleterious [7].

The resolution of organ dysfunction that has been induced by shock, like acute kidney injury, obviously indicates that tissue perfusion occurred. Nevertheless, as stated above, the increase in urine output is unhelpful in the numerous cases in which tubular necrosis has already occurred. Even if this is not the case, the increase in diuresis is very poorly correlated with the simultaneous changes in cardiac output [8]. Moreover, when it occurs, the increase in urine output is delayed compared to the improvement in renal perfusion [9].

In general, looking for the improvement in tissue oxygenation likely requires more sophisticated indices (Fig. 1). In a study performed in critically ill patients, in the subgroup in which cardiac output increased, oxygen consumption improved significantly (by more than 15%) in only 56% of patients [3]. In the other ones, the increase in cardiac output was not accompanied by any beneficial effect on oxygen consumption. In fact, these results are in agreement with the physiology of the relationship between oxygen delivery and consumption. In patients in whom increased cardiac output was accompanied by an increase in oxygen consumption, oxygen consumption and delivery were likely working on the dependent part of their relationship.

These results do not mean that volume expansion was unnecessary in cases where oxygen consumption did not increase. The increase in oxygen delivery profitably moves the patient away from the dangerous zone where oxygen consumption becomes dependent on delivery. Nevertheless, it must be kept in mind that aiming at achieving supranormal values for cardiac output or normal values for mixed venous oxygen saturation does not reduce morbidity or mortality among critically ill patients [7].

**Fig. 2:**
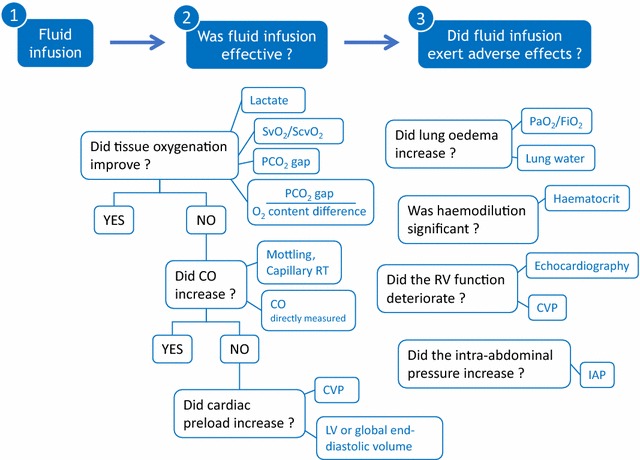
Summary of the criteria on which the benefits and risks of volume expansion might be assessed. *CO* cardiac output, *CVP* central venous pressure, *FiO*_*2*_ oxygen inspired fraction, *IAP* intra-abdominal pressure, *LV* left ventricular, *PCO*_*2*_ carbon dioxide partial pressure, *PaO*_*2*_ arterial oxygen partial pressure, *RT* refill time, *SvO*_*2*_*/ScvO*_*2*_ mixed/central venous oxygen saturation

What these results suggest is that the effects of fluid administration on tissue oxygenation should be monitored. To do this, several indices might be considered. Mixed or central venous saturation of oxygen (SvO_2_ and ScvO_2_, respectively) might be useful. When it is low and when it increases with volume expansion, it means that tissue oxygenation improved [10, 11] (Fig. 2). Nevertheless, if the oxygen delivery/consumption relationship is in its dependent zone, an increase in oxygen delivery does not induce a substantial increase in SvO_2_/ScvO_2_ because oxygen extraction is maximal until oxygen delivery increases above its critical value. Another limitation in SvO_2_/ScvO_2_ might appear in patients with septic shock with tissue oxygen extraction impairment, since the SvO_2_/ScvO_2_ value remains in the normal range and constant in spite of anaerobic metabolism.

Biochemical markers of anaerobic metabolism might be useful in these cases. Lactate, the most commonly used, will decrease if oxygen delivery increases under the effect of fluid. Its main drawback is that its changes are slow. A decrease in the veno-arterial difference in carbon dioxide (CO_2_) pressure (PCO_2_ gap) will indicate that the cardiac output increase is enough to bring more CO_2_ towards the lungs (Fig. 2). Nevertheless, it is not a marker of anaerobic metabolism. By contrast, the ratio of PCO_2_ gap over the arteriovenous difference in oxygen content, an estimate of the respiratory quotient, more directly reflects anaerobic metabolism [12]. It is as reliable as lactate to indicate that tissue oxygenation increases with fluid administration, but its potential advantage is that it will decrease more rapidly [3] (Fig. 2).

### Did cardiac output increase?

If fluid administration has not improved tissue oxygenation, one should check that it has actually increased cardiac output. Indeed, a significant increase in cardiac output in response to fluid administration happens inconstantly because the shape of the Frank–Starling curve, that relies stroke volume and cardiac preload, is flat [13]. This justifies predicting fluid responsiveness before performing volume expansion, but also to assess the fluid response once it has been performed.

The first way to evidence a fluid-induced increase in cardiac output might be to look for the decrease in the sympathetic reflex-induced vasoconstriction in territories which perfusion has been “sacrificed” for the benefit of more vital organs. In practice, one may look for the disappearance of skin mottling or the increase in the capillary refill time. Of course, these signs are unhelpful if they are absent at baseline. Moreover, they are hardly precisely quantified, though methods have been developed for this purpose [14].

In fact, to estimate the effects of fluid infusion on cardiac output, there is no other way than… to measure cardiac output! Unfortunately, simple changes in blood pressure cannot accurately estimate the effects of fluid loading. In a study in which 228 patients received volume expansion, changes in arterial pulse pressure, measured via a femoral catheter, were very roughly correlated with those of cardiac output [4]. The changes in arterial *pulse* pressure detected fluid responders—in terms of cardiac output—with 22% false negatives. The changes in *mean* arterial pressure were even less reliable [4]. In another study, in which arterial pressure was measured at the radial site, there was no correlation at all between changes in arterial pulse pressure and cardiac output [15]. These findings are easily explained by the basic cardiovascular physiology. Mean arterial pressure is regulated by the sympathetic system which tends to keep it constant while cardiac output varies. It is the value of arterial pressure that is the worst to reflect changes in cardiac output, and its changes during volume expansion are systematically damped compared to those of cardiac output. Arterial pulse pressure is physiologically related to stroke volume [16], but this relationship is not linear because it is influenced by the arterial compliance and the pulse wave amplification [4].

In practice, this likely means that in complex patients, in which a precise assessment of treatments effectiveness is mandatory, effects of fluid administration should be monitored by direct measurements of cardiac output. Of note, all the techniques that measure cardiac output are not equivalent for this purpose. Some techniques (pulmonary artery catheter, transpulmonary thermal or lithium dilution devices, oesophageal Doppler and echocardiography) provide a more direct estimation of cardiac output than some other ones (pulse contour analysis, bioreactance). They also differ in terms of precision. The least change in cardiac output that can be deemed as significant is, for instance, as low as 5% for pulse contour analysis, but only 15% when the pulmonary artery catheter, transpulmonary thermodilution devices or echocardiography are used [17].

### If cardiac output did not increase: did cardiac preload increase?

If cardiac output did not increase with fluid infusion, two reasons might be invoked. Either cardiac preload increased but the patient was not preload responsive, or the volume of fluid administered was not enough to significantly increase cardiac preload (Fig. 1) [1]. This may occur if the dose of fluid was too small or if fluid has diluted in a large, dilated, venous compartment.

In order to rule out the latter hypothesis, one may look at the fluid-induced changes in the markers of cardiac preload. Among them, the cardiac end-diastolic volume might be estimated through echocardiography or transpulmonary thermodilution [17]. In theory, they should increase with volume expansion. Nevertheless, one must admit that these measurements are not very precise, such that small but significant increases in cardiac preload might be missed. For instance, the least significant change of transpulmonary thermodilution for measuring the global end-diastolic volume is only 12% [18].

The measurement of central venous pressure is more precise. Nevertheless, central venous pressure does not necessarily increase significantly, even in cases where volume expansion has increased cardiac preload. Indeed, in patients who respond to volume expansion by an increase in cardiac output—and thus in venous return, which equals cardiac output at steady state—the increase in venous return is provoked by an increase in its pressure gradient (mean systemic pressure—right atrial pressure). For this to happen, right atrial pressure—and thus central venous pressure—must increase to a lesser extent than the mean systemic pressure. Thus, in theory, fluid administration might be effective and cardiac output might increase although the central venous pressure did not change to a large extent. This limitation might be overcome by measuring the mean systemic pressure, but this is not possible in routine practice. Nevertheless, it remains that in fluid non-responders, central venous pressure increases as much as the mean systemic pressure. Then, in theory, if cardiac output does not increase with volume expansion, a reliable way to ascertain that this was due to fluid unresponsiveness, and not to an insufficient fluid volume, is to look for an increase in the central venous pressure. Nevertheless, increasing the central venous pressure is not per se a goal of fluid administration.

## Did volume expansion induce adverse side effects?

Like any other drug, whatever its efficacy, the adverse side effects that may result from fluid administration must be carefully searched. Continuously balancing their weight against haemodynamic benefits helps decide to go on with fluid administration or to choose another therapeutic strategy.

### Did lung oedema appear/worsen?

Volume expansion might increase the total cumulative fluid balance in case of renal failure. As it has been now very clearly established, the higher the cumulative fluid balance, the higher the mortality of critically ill patients, especially in case of acute respiratory distress syndrome (ARDS) [19] and/or septic shock [20]. The increase in fluid balance is due both to the facts that copious amounts of fluids are administered and that fluids in excess cannot be normally eliminated through the kidneys. This is due to a potential renal failure and to the accumulation of fluid in the interstitial compartment. This capillary leak syndrome is favoured by systemic inflammation and hypoalbuminemia.

Besides the cumulative fluid balance, more specific signs of fluid overload must be sought. An increase in the peripheral subcutaneous oedema clearly indicates that fluid has leaked out of the vessels. Obviously, the highest risk is to aggravate lung oedema. Chest X-ray is not sensitive enough since it can detect only large increases in lung oedema. High values of central venous pressure or of pulmonary artery occlusion pressure could be more specific. Nevertheless, they do not take into account a key parameter, which is pulmonary capillary permeability. If pulmonary capillary permeability is much increased, lung oedema might have appeared even though the values of pulmonary artery occlusion pressure and central venous pressure are not much increased.

Extravascular lung water, which is the volume of fluid accumulated in the interstitium and alveoli, is a much more direct reflection of lung oedema created by fluid accumulation (Fig. 2). Then, it is likely one of the most meaningful variables of the adverse side effects of volume expansion, especially in patients with ARDS [21]. Extravascular lung water can be estimated at the bedside through transpulmonary thermodilution [22]. This is likely the most interesting aspect of that technique, and the estimation can now be considered as reliable [17, 22]. In a randomised study including critically ill patients, it has been shown that the cumulative fluid balance was better maintained if clinicians guided their fluid strategy by measuring extravascular lung water rather than the pulmonary artery occlusion pressure [23]. This was associated with a decrease in the duration of ventilation and of the stay in the intensive care unit [23].

### Has the right ventricular function deteriorated?

In case of right ventricular failure, as during ARDS, a specific risk of fluid infusion is to aggravate the right ventricular dilation. The right ventricle is very sensitive to changes in its afterload, which explains the risk that it dilates in case of ARDS. In addition, in case of right ventricular preload unresponsiveness, any fluid administration might further increase the right ventricular overload, and finally the right ventricular dilation. The septal shift it induces should reduce the left ventricular filling and contribute to the decrease in cardiac output.

The right ventricular impairment should be assessed if several fluid infusions have been repeated in a few hours. There is no doubt that echocardiography is the gold standard to assess the right ventricular failure. Nevertheless, it cannot be easily repeated over the day. A faster and easier means is to look for increases in the central venous pressure [24]. This emphasises the value of central venous pressure (Fig. 2), which should not be used to decide to give fluid, since it is not a reliable marker of fluid responsiveness, but which is a reliable marker of the right heart function. An elevation in central venous pressure should prompt to confirm the right ventricular dysfunction by echocardiography (Fig. 2).

### Did volume expansion increase the intra-abdominal pressure?

Worsening of prior intra-abdominal hypertension (IAH) is likely one of the adverse effects of fluid resuscitation that is often neglected in practice. One should always keep in mind that the incidence of IAH in critically ill patients is around 20–30% on admission, while as many as 50–70% of patients (depending on the condition) may develop IAH during the first week of stay in the intensive care unit [25].

One of the most important risk factors for IAH is fluid resuscitation [26]. There is a meaningful correlation between IAH, extravascular lung water kinetics and fluid balance in critically ill patients [27]. In fact, IAH may induce organ dysfunction through two major pathways: firstly, by decreasing the perfusion pressure gradient of the intra-abdominal organs; secondly, by impairing systemic haemodynamics. Typically, organ dysfunction appears when intra-abdominal pressure is higher than 20–25 mmHg [25]. This should spur us to assess the effects of volume expansion on the intra-abdominal pressure in patients with suspected or established IAH (Fig. 2).

### Did volume expansion induce haemodilution?

Administration of crystalloids or colloids unavoidably results in haemodilution, and the degree of this haemodilution is far from negligible. One must keep in mind that the normal total blood volume is 65–70 mL/kg and that it is even reduced in many conditions associated with circulatory failure. A volume expansion of even only 500 mL is significant.

In a study in which critically ill patients received a 500-mL volume expansion, our group showed that this resulted in a decrease in haemoglobin concentration by 8%. More importantly, in patients who did not respond to fluid by an increase in cardiac output (non-responders), this led to a significant decrease in oxygen delivery [3]. Clearly, these results once more emphasise how volume expansion is deleterious in fluid non-responders.

## Conclusion

Fluids should be considered as a drug, which positive effects are inconstant and which carries a significant risk of adverse effects. Like for any drug, one should not miss after having administered fluids to ask two questions: has it been effective and has it been harmful? Who would administer antibiotics without assessing the body temperature or the biological markers of inflammation? Who would administer aminosides without checking the renal function? The correct fluid management should not be limited to the prediction of fluid responsiveness, but should include a cautious assessment of fluids benefits and side effects.

## Abbreviations

ARDS: acute respiratory distress syndrome
IAH: intra-abdominal pressure
CO_2_: carbon dioxide
PCO_2_ gap: veno-arterial difference in carbon dioxide tension
SvO_2_/ScvO_2_: central/mixed venous oxygen saturation
